# Cardiovascular Effects of Epinephrine During Experimental Hypothermia (32°C) With Spontaneous Circulation in an Intact Porcine Model

**DOI:** 10.3389/fphys.2021.718667

**Published:** 2021-09-06

**Authors:** Rizwan Mohyuddin, Erik Sveberg Dietrichs, Predip Sundaram, Timofey Kondratiev, Marie Fjellanger Figenschou, Gary C. Sieck, Torkjel Tveita

**Affiliations:** ^1^Anesthesia and Critical Care Research Group, Department of Clinical Medicine, UiT, The Arctic University of Norway, Tromsø, Norway; ^2^Experimental and Clinical Pharmacology Research Group, Department of Medical Biology, UiT, The Arctic University of Norway, Tromsø, Norway; ^3^Center for Psychopharmacology, Diakonhjemmet Hospital, Oslo, Norway; ^4^Division of Surgical Medicine and Intensive Care, University Hospital of North Norway, Tromsø, Norway; ^5^Department of Physiology and Biomedical Engineering, Mayo Clinic, Rochester, MI, United States

**Keywords:** cardiovascular dysfunction, vasopressor, targeted temperature management, rewarming, electrophysiology, ventricular arrhythmias

## Abstract

**Aims:** Rewarming from accidental hypothermia and therapeutic temperature management could be complicated by cardiac dysfunction. Although pharmacologic support is often applied when rewarming these patients, updated treatment recommendations are lacking. There is an underlying deficiency of clinical and experimental data to support such interventions and this prevents the development of clinical guidelines. Accordingly, we explored the clinical effects of epinephrine during hypothermic conditions.

**Materials and methods:** Anesthetized pigs were immersion cooled to 32°C. Predetermined variables were compared at temperature/time-point baseline, after receiving 30 ng/kg/min and 90 ng/kg/min epinephrine infusions: (1) before and during hypothermia at 32°C, and after rewarming to 38°C (*n* = 7) and (2) a time-matched (5 h) normothermic control group (*n* = 5).

**Results:** At 32°C, both stroke volume and cardiac output were elevated after 30 ng/kg/min administration, while systemic vascular resistance was reduced after 90 ng/kg/min. Epinephrine infusion did not alter blood flow in observed organs, except small intestine flow, and global O_2_ extraction rate was significantly reduced in response to 90 ng/kg/min infusion. Electrocardiographic measurements were unaffected by epinephrine infusion.

**Conclusion:** Administration of both 30 ng/kg/min and 90 ng/kg/min at 32°C had a positive inotropic effect and reduced afterload. We found no evidence of increased pro-arrhythmic activity after epinephrine infusion in hypothermic pigs. Our experiment therefore suggests that β₁-receptor stimulation with epinephrine could be a favorable strategy for providing cardiovascular support in hypothermic patients, at core temperatures >32°C.

## Introduction

Guidelines for targeted temperature management (TTM) and therapeutic hypothermia recommend reducing core temperatures to 36–32°C for neuroprotection in comatose patients after resuscitation from sudden cardiac arrest (Nolan et al., 2015). However, after return of spontaneous circulation, these patients often suffer acute heart failure and need inotropic drugs to support inadequate circulatory function (Bernard et al., 2002; Safar and Kochanek, 2002).

Temperatures similar to those used in TTM are regularly observed in victims of accidental hypothermia. These patients often display hypothermia-induced cardiac dysfunction (HCD), associated with reduced cardiac output (CO) during rewarming. In order to elevate low CO, epinephrine has been used in experimental treatment of HCD (Kondratiev and Tveita, 2006; Tveita and Sieck, 2011). Despite its frequent use as an inotropic agent, little is known about the clinical effects of epinephrine (Epi) in TTM patients and victims of accidental hypothermia. Accordingly, guidelines describing the use of inotropic drugs to support cardiovascular function at low core temperatures are lacking evidence-based information. Thus, it is vital to acquire more translational data to understand the pharmacological properties of Epi at low core temperatures.

During normal core temperatures, Epi works as a β-receptor agonist and increases cardiac contractility *via* a G protein – protein kinase A (PKA) signaling pathway, by increasing cyclic adenosine monophosphate (cAMP). In low concentrations, Epi causes peripheral vasodilation, through β_2_-stimulation and elevated cAMP in smooth muscle. At higher concentrations β-adrenergic selectivity is lost, with increasing α-receptor stimulation, causing vasoconstriction (Dietrichs et al., 2016). Several of our *in vivo* rat studies have shown that Epi failed to elevate stroke volume (SV) and cardiac out (CO) during hypothermia and after rewarming. Moreover, it has been reported that inotropic effect of Epi decreases below 34°C, due to relative higher α-receptor than β-receptor response, shown through elevated total peripheral resistance (Kondratiev and Tveita, 2006; Tveita and Sieck, 2011; Dietrichs et al., 2016). We found that low dose (0.125 μg/min) Epi compared to high dose (1.25 μg/min) gave positive inotropic effect during hypothermia (28°C; Tveita and Sieck, 2011). These results in a rat model indicate that hypothermia exerts a negative impact on cardiac inotropy, mediated by the β_1_-receptor pathway, causing a reduced therapeutic interval for beneficial effects of Epi (Dietrichs et al., 2016).

Fundamental species-dependent differences exist in physiology and metabolism, including pharmacokinetics and pharmacodynamics of different drugs (Toutain et al., 2010). In this study, a pig model was chosen due to its close morphologic and physiologic relationship with humans, giving a higher translational potential than previous findings in rodents (Swindle, 1998). Accordingly, the present study aimed to assess whether a low (30 ng/kg/min) and a high (90 ng/kg/min) doses of Epi provide inotropic support at low core temperatures, relevant in TTM and accidental hypothermia. For this purpose, we monitored global O_2_ delivery (DO_2_), O_2_ consumption (VO_2_), and organ blood flow, as well as cardiac electrophysiology during hypothermia (32°C) and rewarming to 38°C, using an intact porcine model with spontaneous circulation throughout the experiment.

## Materials and Methods

### Ethical Approval

The Norwegian National Animal Research Authority approved the experimental protocol. The experiments were conducted in accordance with the Norwegian Regulation on Animal Experimentation and the European Contention for the Protection of Vertebrate Animals used for Experimental and Other Scientific Purposes (European Council, ETS no. 170). The animals received humane care in accordance to the Norwegian Animal Welfare Act.

### Experimental Animals

Fourteen castrated juvenile male NOROC stock pigs weighing 27 ± 5 kg were acclimated to the animal department for 3–5 days before experiments. Two pigs were used for pilot experiments to determine expedient dose of Epi.

### Anesthesia and Instrumentation

Methods for anesthesia, instrumentation, monitoring, sampling, data analysis, and calculations used in the porcine animal model for this study were previously described in detail (Valkov et al., 2019). Briefly, after an overnight fast, the pigs were premedicated by *i.m.* injections of 20 mg/kg ketamine, 30 mg midazolam, and 1 mg of atropine. Anesthesia was induced by intravenous injection of pentobarbital 10 mg/kg and fentanyl 0.01 mg/kg. The pigs were tracheostomized and intubated with a 7.0 mm Portex tracheal tube. They were ventilated with end-expiratory pressure of 0 cm H_2_O, FiO_2_ 0.21 (Siemens Servo 900D, Solna, Sweden). Arterial, central venous, mixed venous, and cerebral venous were sampled and analyzed (ABL800 FLEX, Radiometer medical, Copenhagen, Denmark) to confirm adequate ventilation and calculate global and cerebral O_2_ transport. Anesthesia was maintained by continuous intravenous infusion of pentobarbital 4 mg/kg/h, fentanyl 0.02 mg/kg/h, and midazolam 0.3 mg/kg/h along with infusion of 9 ml/kg/h Ringer-acetate throughout the experiment. Euthanasia was performed by *i.v.* injection of 1 g pentobarbital and 20 ml KCl (1 mmol/ml) at the end of experiment. Medical infusions were provided through an 18 Ga central venous line in the right jugular vein. Cerebral venous blood gases were obtained from a retrograde 18 Ga central venous line in the jugular bulb, introduced through the left internal jugular vein (7–10 cm). Pulmonary artery pressure, central venous pressure (CVP), core temperature, and CO measurements were enabled *via* a 7.5 Fr Swan-Ganz catheter (Edwards Lifesciences, Irvine, United States) in the pulmonary trunk through an 8.5 Fr Intro-Flex introducer (Intro-Flex, Edwards Lifesciences, Irvine, United States) in the left femoral vein. CO was measured by thermodilution using a CO-monitor (Vigilance, Edwards Lifesciences, Irvine, United States). For preload reduction, a 7 Fr Fogarty catheter (Edwards Lifesciences, Irvine, United States) was placed in the inferior vena cava *via* an 8.5 Fr introducer in the right femoral vein. A 7 Fr pressure-volume (PV) catheter (Ventri-Cath-507, Millar Instruments, Huston, TX, United States) was placed in the left ventricle *via* a 10 Fr Super Arrow-Flex introducer (Arrow, Reading, United States) in the right carotid artery. A 7.5 Fr Swan-Ganz catheter was lead through an 8 Fr introducer (Super Arrow-Flex PSI Set, Arrow, Reading, United States) in the left femoral artery and positioned in the aortic arch for measurement of blood pressure, sampling of blood for arterial blood gases and microspheres reference sampling. Injections of stable isotope labeled microspheres were performed *via* a 6 Fr pigtail catheter (Cordis Corporation, Miami, FL, United States) positioned in the left ventricle *via* an 8 Fr introducer through the right femoral artery. All animals received 5000 IU heparin after instrumentation. A 16 Fr Foley catheter (Teleflex Medical, Perak, Malaysia) was introduced *via* a lower abdominal incision for continuous monitoring of urinary output.

### Experimental Protocol

Following surgery, animals were allowed to rest for 30 min before experiments started. To assess the appropriate dose of Epi, a pilot experiment was performed using two pigs. One pig received Epi infusions at normothermia in doses of 15, 30, 45, 60, 75, 90, 105, and 120 ng/kg/min, while monitoring HR, MAP, CVP, and CO. A 25–30% increase in CO was determined as a target for the first Epi dose (E1), whereas the target for the second Epi dose (E2) was inducing a maximal increase of CO. The selected doses were administered again after the initial tests to test the reproducibility of the results. The second pig was cooled to 32°C, and Epi doses of 30, 75, and 90 ng/kg/min were administered to test effect on CO. From these pilot experiments, Epi doses of 30 and 90 ng/kg/min were chosen.

**Figure 1:**
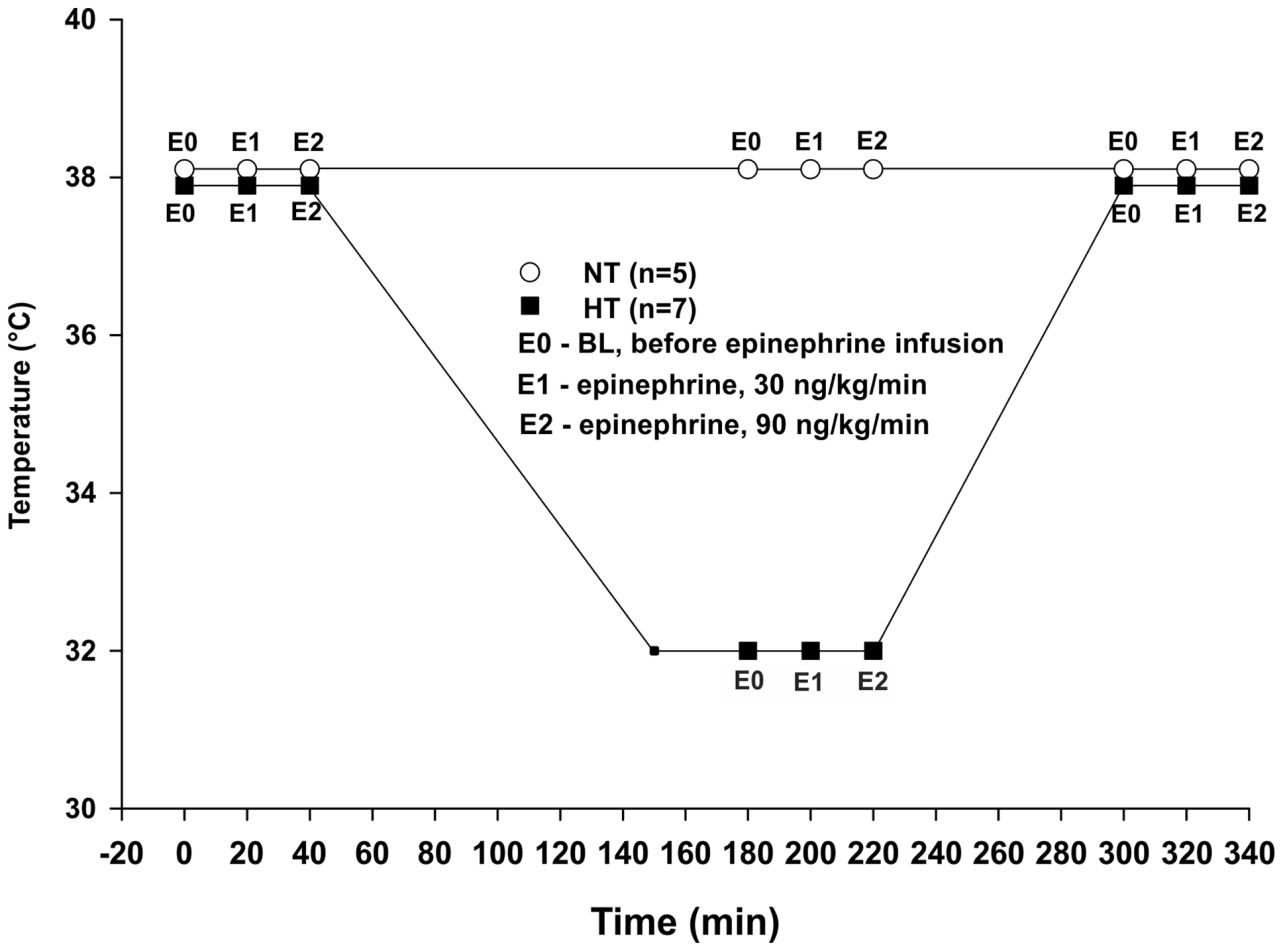
Experimental protocol. NT – time-matched normothermic group, kept at 38°C for 5 h (*n* = 5); HT – hypothermic group, cooled to 32°C and rewarmed back to 38°C (*n* = 7); E0 – no epinephrine; E1 – epinephrine, 30 ng/kg/min; and E2 – epinephrine, 90 ng/kg/min.

Twelve animals were divided into two groups: normothermic (NT group, *n* = 5) and hypothermic (HT group, *n* = 7). Sample size was calculated using power analysis. In both groups, data were assessed at three different time points. For the NT group recordings were performed at baseline after initial stabilization, and then after 3 h and 5 h at 38°C (Figure 1). For the HT group, recordings were performed at 38°C baseline, and then after 30 min and 3?? at 32°C (3 h) and also following rewarming to 38°C (5 h). At each time period, three samples were obtained as: baseline before starting Epi infusion (E0), during 30 ng/kg/min Epi infusion (E1), and during 90 ng/kg/min Epi infusion (E2). Each Epi infusion continued for at least 5 min before recording was started, and lasted 15–20 min. All hemodynamic data were recorded using a digital acquisition system (PowerLab 16/35, ADInstruments, Bella Vista, Australia and MPVS Ultra PV unit, Millar Instruments, Huston, TX, United States) and were analyzed using Lab Chart Pro 8 software (ADInstruments, Bella Vista, Australia).

### Temperature Regulation

Core body temperature was monitored *via* a thermistor positioned at the tip of the thermodilution catheter positioned in the pulmonary artery. Animals in the HT group were cooled by circulating iced water in a reservoir mounted to the top of the operating table with the animals placed in a supine position, leaving the dorsal 2/3 of the body submerged. As blood temperature reached 32°C, water was drained from the reservoir and temperature stabilized at 32 ± 1°C, which was chosen due to this temperature being the lower end of therapeutic temperature management in comatose survivors of cardiac arrest. For rewarming, the reservoir was circulated with tap water at a temperature of 38–40°C.

### Regional Blood Flow

Regional blood flow was estimated using method of neutron activation of stable isotope labeled microspheres (BioPAL Inc., Worchester, MA, United States) proposed by Reinhardt et al. (2001) as a high-sensitive and reliable method for measuring of regional blood flow in intact tissue. This method was refined to use in our animal model as described in detail by Valkov et al. (2019).

At every data sampling time point an injection of ∼ 10^6^ microspheres, of different specificity, was given through a fluid-filled pigtail catheter placed in the left ventricle. At the same time, a reference blood sample was drawn from the aortic arch using a withdrawal pump set at a fixed sample rate (5 ml min^−1^) for 2 min (New Era Pump Systems, Inc., Farmingdale, NY, United States). Each reference blood sample, with the predetermined type of microsphere in accordance with the data collection point, was collected in 20 ml sample vials. The syringe was rinsed with “sansSaLine” medium (BioPAL, Inc.) to secure removal of microspheres attached to the wall. Reference blood samples were centrifuged twice with “sansSaLine” to remove sodium and chloride together with the supernatant to improve the signal-to-noise ratio of the sample. After the end of the experiment, animals were killed, and organ tissue samples were taken from the same locations in all animals and rinsed with “sansSaLine” to remove surface blood and other potential contaminants. Both reference blood samples and organ tissue samples were dried in an oven (70°C overnight). After processing, reference blood samples and tissue samples were analyzed at the BioPAL laboratory for specific activity. Tissue samples for evaluation of regional blood flow were taken from the heart, brain, kidneys, liver, stomach, and small intestine. Determination of regional tissue blood flow (expressed in milliliters per minute per gram) was calculated using the following equation: $Q=\left({\mathrm{Tis}}_{\mathrm{dpm}}\times Q_{\mathrm{ref}}\right)/\left({\mathrm{Ref}}_{\mathrm{dpm}}\times g\right)$, where *Q* is blood flow in milliliters per minute, Tis_dpm_ is the number of radioactive decays in the tissue sample in decays per minute, *Q*_ref_ is the reference flow rate in milliliters per minute, Ref_dpm_ is the number of radioactive decays in the reference blood sample in decays per minute, and *g* is weight of the tissue sample in grams (Valkov et al., 2019).

### Hemodynamic Variables, Global and Cerebral O_2_ Transport, and O_2_ Extraction Rate

Left ventricular pressure was measured by a pressure transducer on the tip of the PV catheter. Left ventricular contractility was assessed by occluding the inferior vena cava with an inflatable balloon. At each data sampling point, the balloon was inflated to obtain different levels of mechanical work. To relate the raw measurement to an estimate of actual volume, transthoracic echocardiographic measurements (parasternal short axis view) of end diastolic diameter and end systolic diameter were obtained at all data sampling points. Using the Teichholz formula $\left(\mathrm{EDV}=\left[\frac{7}{2.4+\mathrm{EDD}}\right]\times {\mathrm{EDD}}^{3}\right)$, left ventricular end diastolic volume (EDV) and end systolic volume were calculated. The same operator conducted all echocardiographic measurements.

CO values were obtained from thermodilution data. Stroke volume (SV) was calculated as: SV = CO/HR. O_2_ content (ContO_2_) values in arterial, mixed venous, and jugular bulb samples were calculated according to the formula: ${\mathrm{ContO}}_{2}=\left({\mathrm{sO}}_{2}\times \mathrm{Hb}\times \left(1.34\times 10^{-2}\right)\right)+\left({\mathrm{pO}}_{2}\times 0.023\right)$, where sO_2_ is blood O_2_ saturation (%) and Hb is hemoglobin (ml/dl). Global DO_2_ was calculated as the product of CO and arterial ContO_2_ per kg body weight. Global VO_2_ was calculated as the product of CO and the difference between arterial and mixed venous ContO_2_ per kg body weight. Cerebral DO_2_ was calculated as a product of arterial ContO_2_ and cerebral blood flow (CBF). CBF was estimated using the average flow of the five cerebral samples (right and left temporal, right and left cerebellum, and hippocampus) using the regional blood flow estimation technique. Cerebral VO_2_ was calculated as a product of difference between arterial and jugular bulb ContO_2_ and CBF. Global and cerebral O_2_ extraction rate (O_2_ ER) was calculated as the ratio of corresponding VO_2_ to DO_2_ values.

### Electrocardiogram

Internal resultant ECG signals from the conductance catheter were used. The first 4–5 beats in steady state at each recording point were averaged and analyzed using Lab Chart Pro 8 software. QT interval was corrected for HR using Bazett’s formula (QTc). QRS/QTc was calculated according to a recent publication relating this parameter to risk for hypothermia-induced cardiac arrest (Dietrichs et al., 2020a,b).

### Statistics

Sample size was calculated using power analysis. The results for blood flow and O_2_ transport are presented as means ± SD. The results for hemodynamic variables and ECG intervals are presented as median and percentiles. Data distribution was assessed using Shapiro-Wilk test. Dependent on data distribution, one-way RM ANOVA for normal distributed data or RM ANOVA on ranks for non-normal distributed data, was used to compare values within groups. If the *F* value was greater than critical, *post-hoc* tests were used to obtain *p* values. *Tukey’s* test was used for pairwise multiple comparison of corresponding values before and during epinephrine infusions within groups. *Dunnett’s* test was used to compare baseline values at 38°C to baseline values before Epi infusion after 3–5 h (NT group) and at 32–38°C after rewarming (HT group). The values of *p* < 0.05 were considered statistically significant.

## Results

### Cooling and Rewarming Rates

All animals survived the experimental protocol. Cooling from 38°C to 32°C was carried out with a cooling rate of 4°C/h, while the rewarming rate was 4.5°C/h.

### Responses to Epi Infusion in NT and HT Groups at 38°C Baseline

#### Hemodynamics

**Figure 2:**
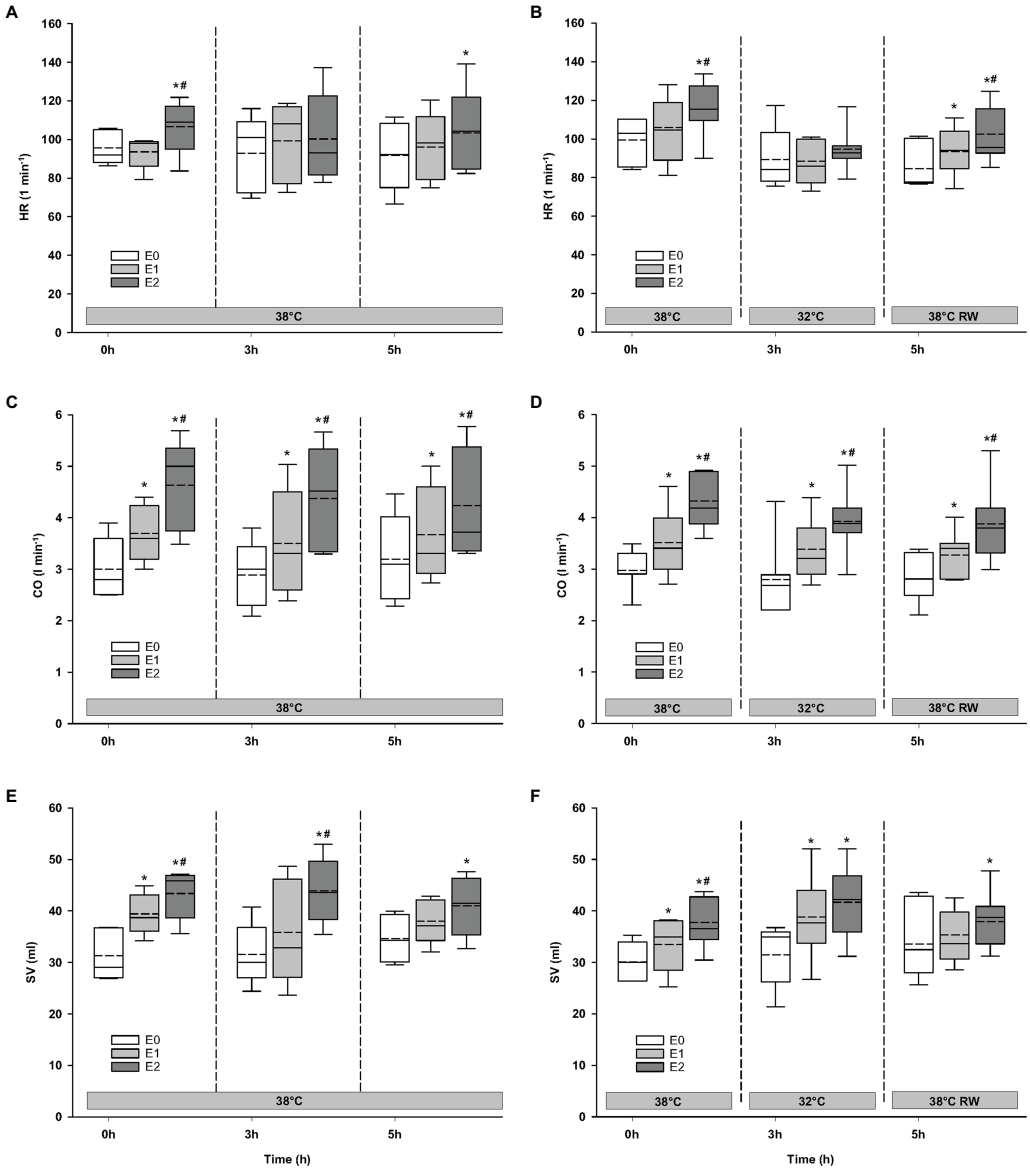
Hemodynamic variables. **(A,B)** HR, heart rate; **(C,D)** CO, cardiac output; and **(E,F)** SV, stroke volume. *N =* 5 (NT group, left column) and *n* = 7 (HT group, right column). E0 – before epinephrine infusion, E1 – epinephrine infusion 30 ng/kg/min, E2 – epinephrine infusion 90 ng/kg/min, and RW – rewarming. Data are presented as vertical boxes with median (solid line), mean (dashed line), and interquartile range with 10th and 90th percentile error bars. Significance is indicated by: ^*^ – compared with corresponding E0 and ^#^ – compared with corresponding E1 (30 ng/kg/min). Level of significance: *p* < 0.05.

**Figure 3:**
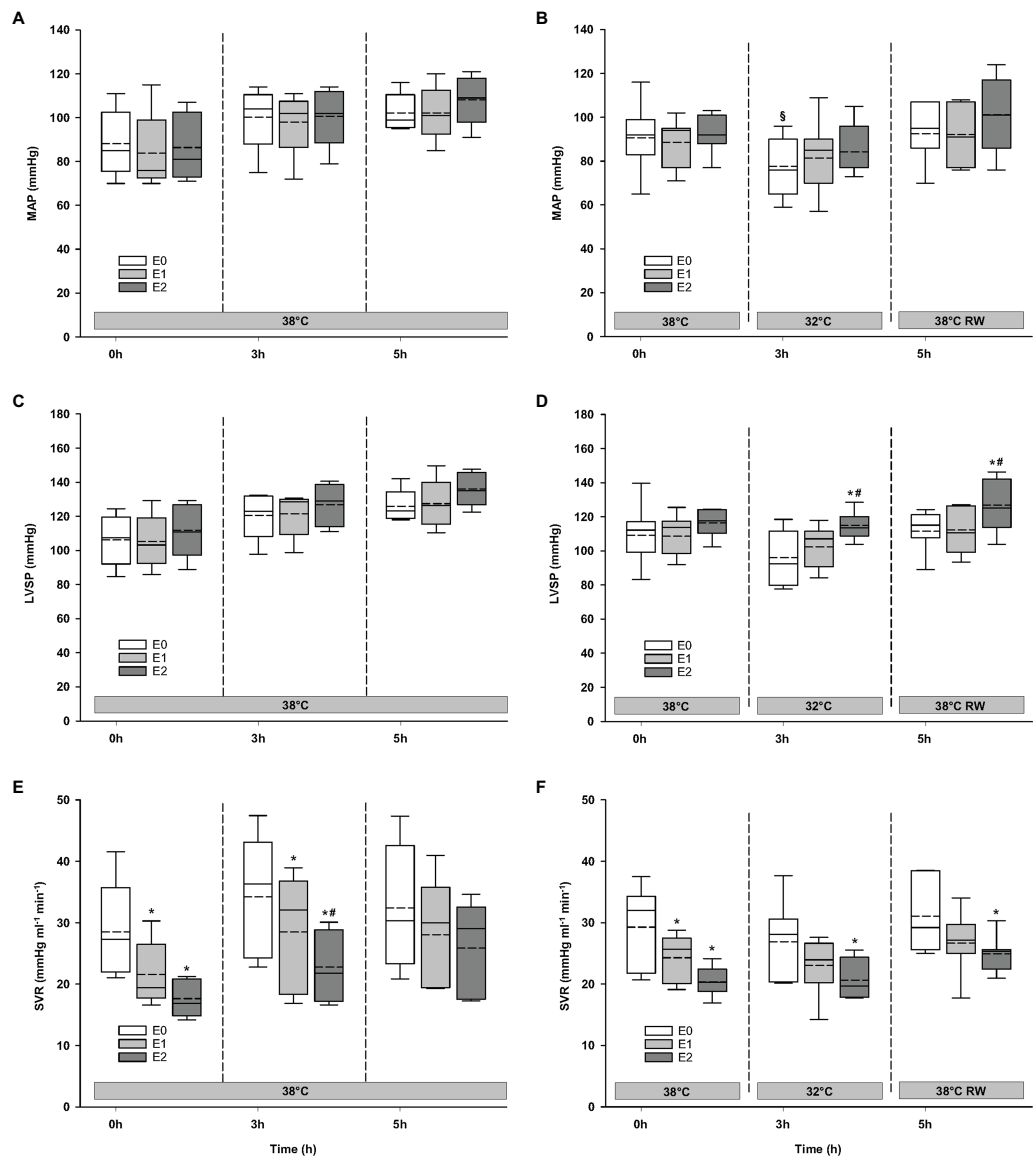
Hemodynamic variables (cont.). **(A,B)** MAP, mean arterial pressure; **(C,D)** LVSP, left ventricular systolic pressure; and **(E,F)** SVR, systemic vascular resistance. *N* = 5 (NT group, left column) and *n* = 7 (HT group, right column). E0 – before epinephrine infusion, E1 – epinephrine infusion 30 ng/kg/min, E2 – epinephrine infusion 90 ng/kg/min, and RW – rewarming. Data are presented as vertical boxes with median (solid line), mean (dashed line), and interquartile range with 10th and 90th percentile error bars. Significance is indicated by: ^*^ – compared with corresponding E0, ^#^ – compared with corresponding E1 (30 ng/kg/min), and ^§^ – compared with absolute (0 h) baseline (E0). Level of significance: *p* < 0.05.

**Figure 4:**
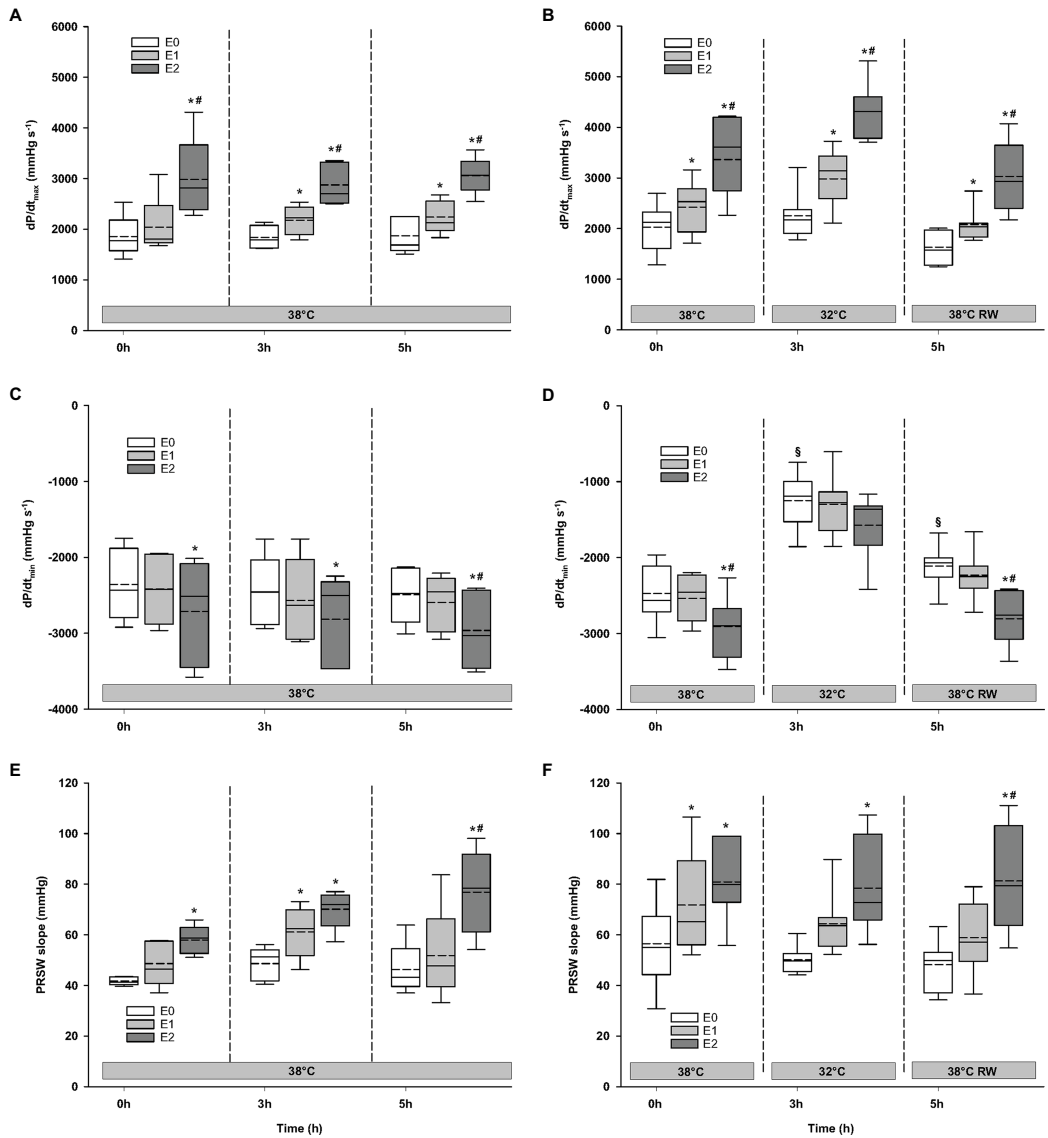
Hemodynamic variables (cont.). **(A,B)** *dP*/*dt*_max_, maximum rate of LV pressure rise; **(C,D)** *dP*/*dt*_min_, maximum rate of LV pressure decline; and **(E,F)** PRSW, preload recruitable stroke work. *N* = 5 (NT group, left column) and *n* = 7 (HT group, right column). E0 – before epinephrine infusion, E1 – epinephrine infusion 30 ng/kg/min, E2 – epinephrine infusion 90 ng/kg/min, and RW – rewarming. Data are presented as vertical boxes with median (solid line), mean (dashed line), and interquartile range with 10th and 90th percentile error bars. Significance is indicated by: ^*^ – compared with corresponding E0, ^#^ – compared with corresponding E1 (30 ng/kg/min), and ^§^ – compared with absolute (0 h) baseline (E0). Level of significance: *p* < 0.05.

Compared to baseline values (before Epi infusion), hemodynamic responses were identical in the two groups (Figures 2–4). A significant increase in HR was observed only in response to 90 ng/kg/min, while SV and CO increased significantly in response to 30 ng/kg/mg. MAP and left ventricular systolic pressure (LVSP) remained unchanged, whereas SVR was significantly reduced in response to 30 ng/kg/min. Both maximum rate of LV pressure rise (*dP*/*dt*_max_) and preload recruitable stroke work (PRSW) increased in response to 90 ng/kg/min.

#### O_2_ Transport

**Figure 5:**
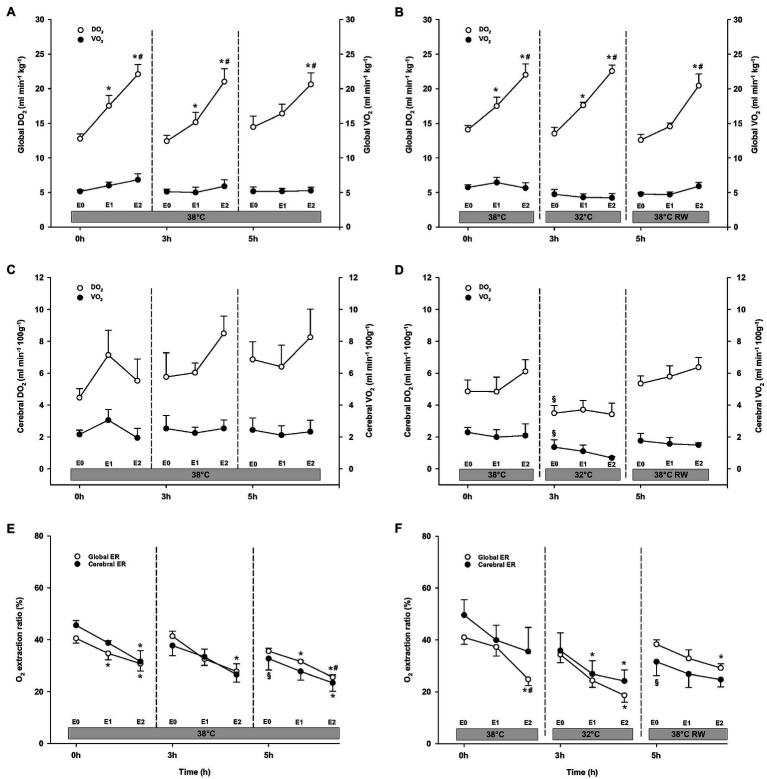
Oxygen transport. **(A,B)** Global oxygen delivery, DO_2_ and consumption, VO_2_; **(C,D)** cerebral oxygen delivery, DO_2_ and consumption, VO_2_; and **(E,F)** global and cerebral O_2_ extraction ratio, O_2_ ER. *N* = 5 (NT group, left column) and *n* = 7 (HT group, right column). E0 – before epinephrine infusion, E1 – epinephrine infusion 30 ng/kg/min, E2 – epinephrine infusion 90 ng/kg/min, and RW – rewarming. Data are presented as means and standard deviation. Significance is indicated by: ^*^ – compared with corresponding E0, ^#^ – compared with corresponding E1 (30 ng/kg/min), and ^§^ – compared with absolute (0 h) baseline (E0). Level of significance: *p* < 0.05.

A similar increase in global DO_2_ was measured in both groups (Figure 5). Global DO_2_ increased in response to 30 ng/kg/min and was further increased by 90 ng/kg/min. Global VO_2_ remained unchanged. Global O_2_ ER was well below the critical value (0.7). A significant reduction was observed in response to 90 ng/kg/min.

#### Organ Blood Flow

**Figure 6:**
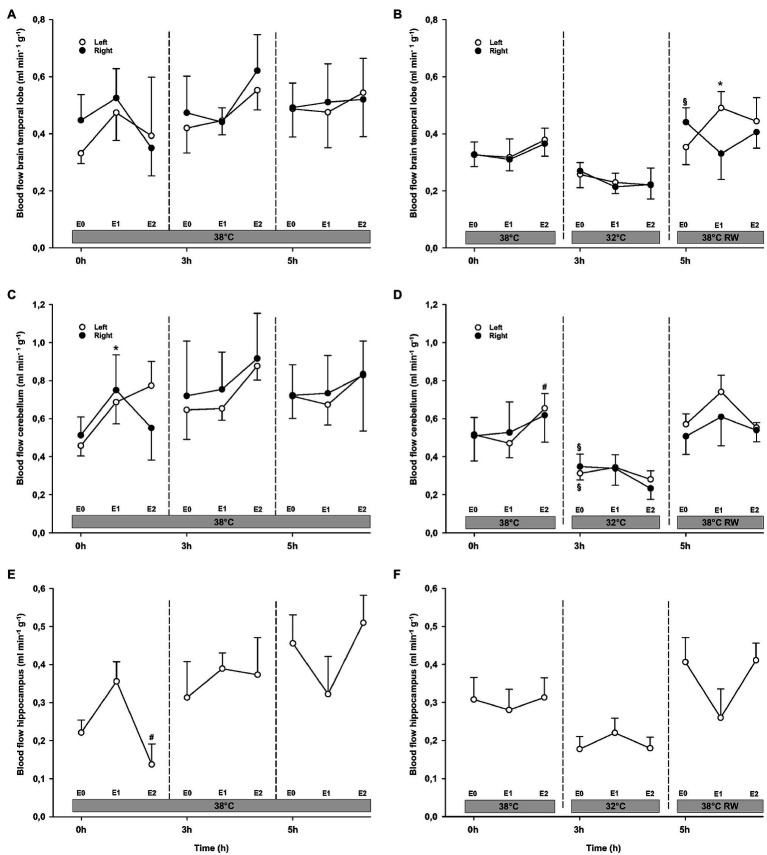
Organ blood flow. **(A,B)** Brain temporal lobe, **(C,D)** cerebellum, and **(E,F)** hippocampus. *N* = 5 (NT group, left column) and *n* = 7 (HT group, right column). E0 – before epinephrine infusion, E1 – epinephrine infusion 30 ng/kg/min, E2 – epinephrine infusion 90 ng/kg/min, and RW – rewarming. Data are presented as means and standard deviation. Significance is indicated by: ^*^ – compared with corresponding E0, ^#^ – compared with corresponding E1 (30 ng/kg/min), and ^§^ – compared with absolute (0 h) baseline (E0). Level of significance: *p* < 0.05.

**Figure 7:**
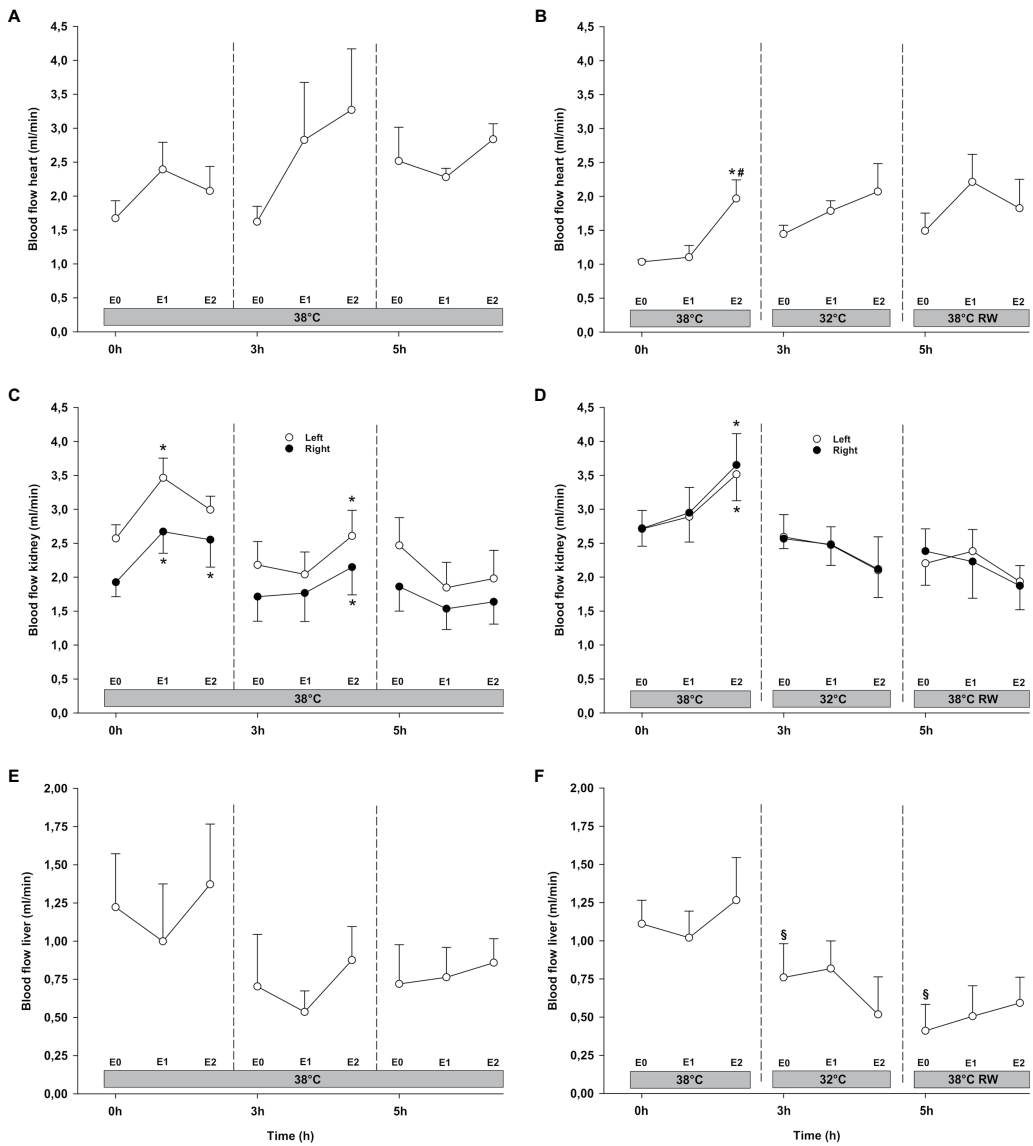
Organ blood flow (cont.). **(A,B)** Heart, **(C,D)** kidneys, and **(E,F)** liver. *N* = 5 (NT group, left column) and *n* = 7 (HT group, right column). E0 – before epinephrine infusion, E1 – epinephrine infusion 30 ng/kg/min, E2 – epinephrine infusion 90 ng/kg/min, and RW – rewarming. Data are presented as means and standard deviation. Significance is indicated by: ^*^ – compared with corresponding E0, ^#^ – compared with corresponding E1 (30 ng/kg/min), and ^§^ – compared with absolute (0 h) baseline (E0). Level of significance: *p* < 0.05.

Epi infusion did not result in any significant changes in regional blood flow in temporal lobes, liver, and gastro-intestinal organs, except small intestine flow at 32°C (not shown; Figures 6, 7). Some blood flow changes occurred in the cerebellum, hippocampus, and heart in response to Epi infusion. These occurred irregularly and were not always in a dose-dependent pattern. Importantly, blood flow in these organs was not reduced during Epi infusion, except for hippocampal blood flow was reduced in the NT group in response to 90 ng/kg/min compared to 30 ng/kg/min. Kidney blood flow was increased after 90 ng/kg/min.

#### Electrocardiogram

**Figure 8:**
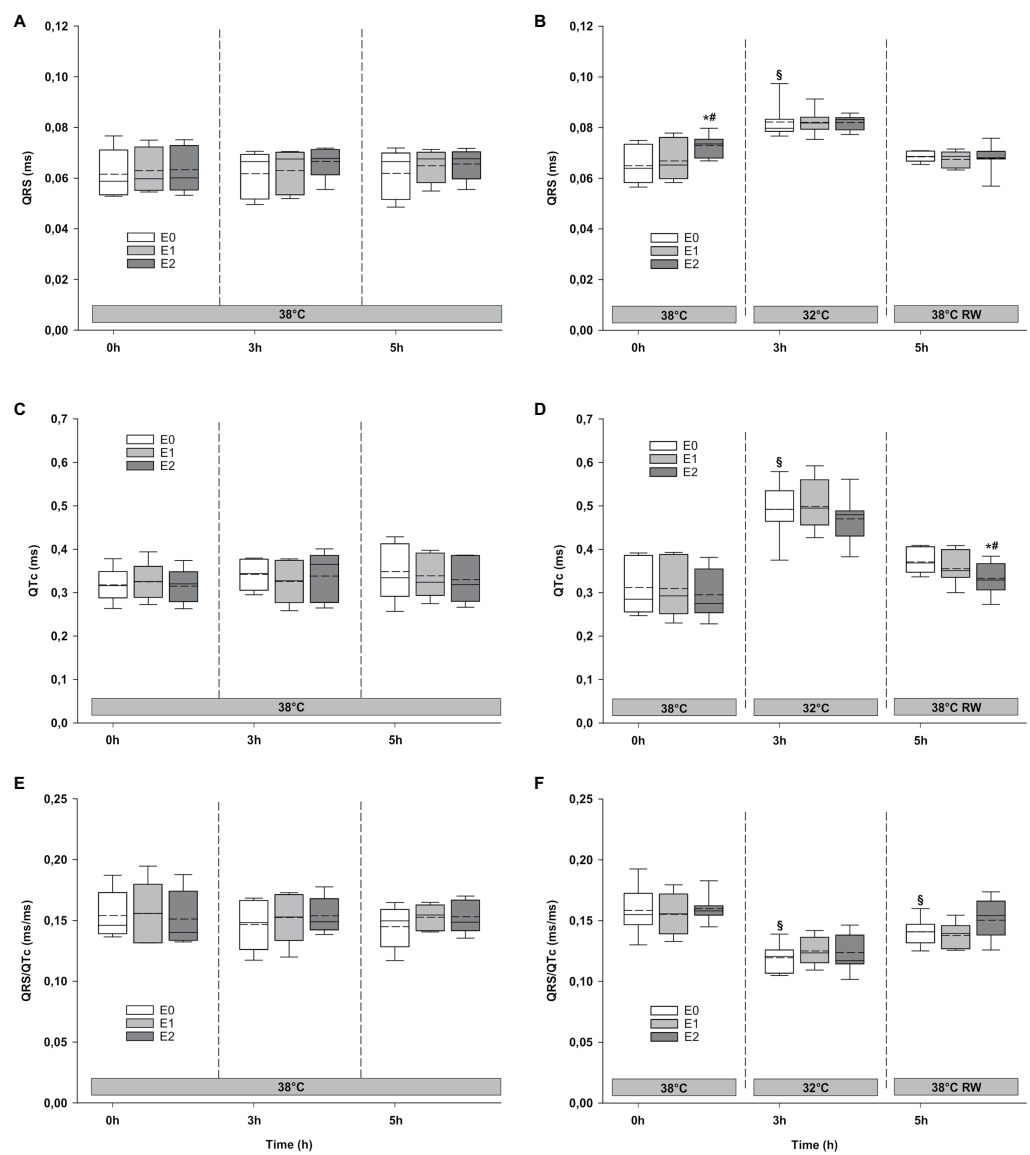
ECG. **(A,B)** QRS interval, **(C,D)** QTc interval, and **(E,F)** QRS/QTc ratio. *N* = 5 (NT group, left column) and *n* = 7 (HT group, right column). E0 – before epinephrine infusion, E1 – epinephrine infusion 30 ng/kg/min, E2 – epinephrine infusion 90 ng/kg/min, and RW – rewarming. Data are presented as vertical boxes with median (solid line), mean (dashed line), and interquartile range with 10th and 90th percentile error bars. Significance is indicated by: ^*^ – compared with corresponding E0, ^#^ – compared with corresponding E1 (30 ng/kg/min), and ^§^ – compared with absolute (0 h) baseline (E0). Level of significance: *p* < 0.05.

Epi infusions did not change duration of QTc intervals, or QRS/QTc ratio (Figure 8). QRS interval increased in the HT group in response to 90 ng/kg/min.

### Responses to Epi Infusion in NT Group at 38°C After 3 h and HT Group at 32°C After 3 h

#### Hemodynamic Variables

For the majority of hemodynamic variables, cooling did not induce significant changes between NT baseline and 32°C (Figures 2–4). Exceptions were MAP and *dP*/*dt*_min_. Neither HR nor MAP was responsive to Epi infusion. In the HT group, LVSP increased during 90 ng/kg/min and CO was elevated in response to 30 ng/kg/min. SV was increased by 30 ng/kg/min in the HT group but only by 90 ng/kg/min in the NT group. Likewise, Epi dose-dependent reduction of SVR was observed in NT group, while in the HT group, SVR was only reduced in response to 90 ng/kg/min. Effect of Epi infusion on *dP*/*dt*_max_ was similar in both groups, with increase in response to 30 ng/kg/min. *dP*/*dt*_min_ was changed only in the NT group after 90 ng/kg/min. PRSW increased in response to 30 ng/kg/min in the NT group, while only elevated by 90 ng/kg/min in the HT group.

#### O_2_ Transport

DO_2_, VO_2_, and O_2_ ER remained unchanged at baseline in the NT group after 3 h (Figure 5). In the HT group, both cerebral DO_2_ and VO_2_ were reduced compared to prehypothermic baseline. Both groups showed dose-dependent increase in global DO_2_ during Epi infusion. However, cerebral O_2_ transport (cerebral DO_2_ and VO_2_) and global VO_2_ were not affected by Epi infusion. Global O_2_ ER was significantly reduced in both groups in response to 90 ng/kg/min. Cerebral O_2_ ER was reduced only in the HT group, after 30 ng/kg/min administration.

#### Organ Blood Flow

Cerebellar and liver blood flow was reduced at 32°C in the HT group, compared to prehypothermic baseline (Figures 6, 7). Epi infusion did not induce effects on blood flow, except for increased kidney blood flow after 90 ng/kg/min in the NT group, and reduced small intestine blood flow after 90 ng/kg/min in the HT group.

#### Electrocardiogram

Cooling resulted in prolongation of QRS and QTc intervals at baseline, while QRS/QTc decreased (Figure 8). Epi infusion did not have any effect on these variables.

### Responses to Epi Infusion in NT Group at 38°C After 5 h and HT Group After Rewarming

#### Hemodynamic Variables

Only *dP*/*dt*_min_ in the HT group changed (decreased) compared to its prehypothermic value (Figures 2–4). In the NT group, HR increased in response to 90 ng/kg/min, whereas in the HT group, Epi elevated HR after 30 ng/kg/min. CO increased in a dose-dependent manner from 30 ng/kg/min in both groups, while SV only increased in response to 90 ng/kg/min. MAP remained unresponsive to Epi in both groups. LVSP was unaffected of Epi infusion in NT group only but increased after 90 ng/kg/min in the HT group. SVR reduction in response to Epi occurred only in the HT group during 90 ng/kg/min. In both groups, *dP*/*dt*_max_ increased after 30 ng/kg/min, while *dP*/*dt*_min_ and PRSW were increased only after 90 ng/kg/min.

#### O_2_ Transport

Compared to their baseline values, cerebral O_2_ ER after 5 h baseline values was significantly reduced in both groups (Figure 5). All other O_2_ transport variables were unchanged. In both groups, 90 ng/kg/min caused an increase in global DO_2_, while global VO_2_, as well as cerebral DO_2_ and VO_2_ remained unaltered by Epi infusion. Global O_2_ ER was reduced in response to 30 ng/kg/min in the NT group, whereas only after 90 ng/kg/min in the HT group. Cerebral O_2_ ER was reduced in response to 90 ng/kg/min in the NT group and remained unchanged in the HT group.

#### Organ Blood Flow

Except for increased baseline blood flow in the right temporal lobe and reduced liver blood flow of the HT group, no changes occurred from baseline (Figures 6, 7). Epi infusions did not alter blood flow in any organ, except for the left temporal lobe in the HT group, where it was increased in response to 30 ng/kg/min.

#### Electrocardiogram

Only QRS/QTc ratio in the HT group was significantly reduced after rewarming compared to prehypothermic baseline (Figure 8). Epi infusion did not affect ECG parameters in the NT group. In the HT group, Epi infusion shortened the QTc intervals in response to 90 ng/kg/min. QRS interval and QRS/QTc ratio remained unaffected by Epi.

## Discussion

This study demonstrated that low- (30 ng/kg/min) and high- (90 ng/kg/min) dose Epi infusion provided positive inotropic effects, both after cooling to 32°C and subsequent rewarming to 38°C. We did not find any indication that Epi infusion increased risk for ventricular arrhythmias during hypothermia.

Our previous studies using an intact rodent model have indicated that the beneficial hemodynamic effects of Epi are lost during cooling to 34°C, as administration gave a detrimental elevation of SVR during hypothermia (Kondratiev and Tveita, 2006; Tveita and Sieck, 2011; Dietrichs et al., 2016). Accordingly, a pharmacodynamic shift toward increased α-receptor-mediated vasoconstriction, over the β-receptor mediated vasodilation and positive inotropic effects observed in normothermic controls, seems to exist in hypothermic rodents, even when administering low doses of Epi. These changes were found at core temperatures regularly observed in victims of accidental hypothermia and as induced in TTM-treated patients. Rewarming from accidental hypothermia is often complicated by HCD, while TTM-patients often suffer acute heart failure and need pharmacological support of cardiovascular function (Bernard et al., 2002; Safar and Kochanek, 2002). In such patients, isolated α-receptor agonist effect of Epi infusion could be treacherous, as afterload increases without simultaneous support of a failing heart. In the present experiment, however, both doses of Epi reduced SVR and had no significant effect on MAP, indicating that the cardiac and peripheral β-adrenergic effects were dominant of these doses, even during hypothermia. This study therefore provides vital information, indicating that the translational potential of earlier rodent findings is limited and that Epi treatment could be suitable in hypothermic patients at core temperatures ≥32°C. As the pigs in the current study were not cooled to temperatures associated with HCD and did not have underlying cardiac disease, the present result advocates further studies exploring the effects of Epi during such pathophysiological conditions in pig.

In the present experiment, Epi infusion induced a dose-dependent increase in PRSW, CO, and SV, both at 32°C and after rewarming. This indicates an intact effect of increasing cardiac inotropy through stimulating the β_1_-receptor – adenyl cyclase (AC) – cyclic AMP (cAMP) – PKA pathway during hypothermia. In rat studies, we found myocardial Ca^2+^ overload and increased cardiac troponin I phosphorylation in relation to HCD (Wold et al., 2013; Schaible et al., 2016). In a failing heart, PKA-mediated phosphorylation of regulatory proteins would expectedly enhance both findings and have a negative impact on cardiac function. The present experiment shows that cardiomyocytes of pigs cooled to 32°C instead have a beneficial response to stimulation of the β_1_-receptor pathway, similar to normothermic animals. Moreover, we find no indication of a pharmacodynamic shift toward an increased peripheral α-receptor response and vasoconstriction, over smooth muscle β_2_-receptor stimulation and vasodilation. The dose-dependent Epi-induced reduction in SVR during hypothermia and rewarming is similar to that in normothermic controls. Accordingly, high-dose Epi infusion did not increase afterload, in stark contrast to rodent findings during deep hypothermia (Dietrichs et al., 2015).

TTM and therapeutic hypothermia are used in different patient groups for neuroprotection after a hypoxic insult, or prior to surgery with reduced cerebral perfusion (Fawaz and Tutunji, 1960; Dietrichs and Dietrichs, 2015). Increased VO_2_ is a well-known effect of Epi infusion (Fawaz and Tutunji, 1960) and could therefore be hypothesized to have a negative impact in hypothermic patients. However, there is no indication that hypoxia is part of the pathophysiology underlying HCD (Kondratiev et al., 2006). Still, Epi infusion could be anticipated to alter organ blood flow, through unbalanced stimulation of peripheral α- and β_2_-receptors in arterioles. Yet, neither hippocampal, cerebellar nor temporal lobe blood flow is changed by Epi infusion during hypothermia. This is accompanied by absence of increased O_2_ demand during drug infusion at 32°C. High-dose Epi increased global DO_2_ in presence of unaltered global VO_2_, both during hypothermia and after rewarming, causing global as well as cerebral O_2_ ER to decrease with Epi infusion during hypothermia. Accordingly, Epi infusion had a positive impact on the ratio between cerebral supply and demand of O_2_ at 32°C. Our findings therefore indicate that use of Epi to support cardiovascular function is suitable during TTM or hypothermia, as there is no suggestion of negative impact of Epi on the neuroprotective effect of core temperature reduction.

Reduction of core temperature is associated with changes in cardiac electrophysiology that increase risk for ventricular arrhythmias, which together with HCD contribute to high mortality of accidental hypothermia (van der Ploeg et al., 2010). The pathophysiological mechanism is disputed with much emphasis on observed J-waves that do not appear to be pathognomonic for hypothermia-induced cardiac arrest (Dietrichs et al., 2019, 2020a). Recent studies find that dispersion of repolarization and a depolarization/repolarization mismatch is associated with increased risk for ventricular fibrillation at 31–32°C (Piktel et al., 2010; Dietrichs et al., 2020a). These pro-arrhythmic changes can be detected by low values of a novel ECG marker for hypothermia-induced cardiac arrest; QRS/QTc (Dietrichs et al., 2020b, 2021; Ref). Hypothermic pigs in the present study were cooled to the same temperature and given β_1_-receptor stimulating Epi infusions.

Due to risk for ventricular arrhythmias, Epi is considered to be unfavorable in repolarization disturbances like long-QT syndrome (Tan et al., 2014), which is a similar to the observed effects of hypothermia on cardiac electrophysiology. In the present experiment, we observed a pronounced prolongation of the QTc interval and a parallel reduction in QRS/QTc values, indicating that cooling had a heterogenic effect on repolarization and depolarization. In rabbit hearts cooled to 31°C, a similar reduction in QRS/QTc values correlated with a 2-fold increase in risk for induction of ventricular fibrillation (Dietrichs et al., 2020a,b). In the present experiment, pigs were probably at a higher risk for developing ventricular arrhythmias at 32°C, compared to at 38°C before Epi infusion. Interestingly, neither low- or high-dose Epi appeared to increase risk for hypothermia-induced arrhythmias, as QRS/QTc values remained stable throughout infusions at 32°C. Thus, the electrophysiological effects of Epi do not appear to prevent utilization of the positive hemodynamic properties of drug infusion during hypothermia and rewarming, as shown in this study.

## Conclusion

Administration of both 30 ng/kg/min and 90 ng/kg/min Epi infusion during hypothermia (32°C) produced a positive inotropic effect and reduced afterload in a porcine model of hypothermia and rewarming. The observed hemodynamic response corresponded with findings in the normothermic control group, and are different from earlier reports, reporting negative hemodynamic impact of catecholamine administration in hypothermic rodents. Further, we find no evidence of increased pro-arrhythmic activity after Epi infusion in hypothermic pigs. The results of these experiments therefore suggest that β₁-receptor stimulation with epinephrine is a favorable strategy for providing cardiovascular support in hypothermic patients, at core temperatures >32°C.

## Data Availability Statement

The raw data supporting the conclusions of this article will be made available by the authors, without undue reservation.

## Ethics Statement

The animal study was reviewed and approved by the Norwegian National Animal Research Authority.

## Author Contributions

TT, GS, RM, ED, and TK contributed to the conception and design, the data analysis and interpretation, and revision of the manuscript. RM, PS, TK, and ED contributed to the completion of experiments and collection of the data. TT, GS, RM, ED, TK, MF, and PS contributed to drafting of the manuscript for intellectual content. All authors contributed to the article and approved the submitted version.

## Conflict of Interest

The authors declare that the research was conducted in the absence of any commercial or financial relationships that could be construed as a potential conflict of interest.

## Publisher’s Note

All claims expressed in this article are solely those of the authors and do not necessarily represent those of their affiliated organizations, or those of the publisher, the editors and the reviewers. Any product that may be evaluated in this article, or claim that may be made by its manufacturer, is not guaranteed or endorsed by the publisher.
